# Patient-reported outcome measures for pain in women with pelvic floor disorders: a systematic review

**DOI:** 10.1007/s00192-022-05126-4

**Published:** 2022-03-02

**Authors:** Maisie Ralphsmith, Susannah Ahern, Joanne Dean, Rasa Ruseckaite

**Affiliations:** Department of Epidemiology and Preventive Medicine, Monash University, Melbourne, Victoria 3004 Australia

**Keywords:** Pain, Patient-reported outcome measures, Pelvic floor disorder, Public health, Registry

## Abstract

**Introduction and hypothesis:**

Patient-reported outcome measures (PROMs) are helpful instruments when measuring and reporting changes in patient health status (Al Sayah et al. J Patient Rep Outcomes 5 (Suppl 2):99, 2021) such as the health-related quality of life (HrQoL) of women with pelvic organ prolapse (POP) and stress urinary incontinence (SUI). The Australasian Pelvic Floor Procedure Registry (APFPR) aims to increase capacity for women to report surgical outcomes through the collection of HrQoL data (Ruseckaite et al. Qual Life Res. 2021) but currently lacks a pain-specific PROM for women with pelvic floor disorders (PFDs), particularly POP and SUI. This review aims to systematically review the existing literature and identify instruments that measure pain in women with POP and SUI for inclusion within the APFPR, which reports on complications from these conditions.

**Methods:**

We conducted a literature search on OVID MEDLINE, Embase, CINAHL, PsycINFO and EMCARE databases in addition to Google Scholar and grey literature to identify studies from inception to April 2021. Full-text studies were included if they used PROMs to measure pain in women with POP and SUI. Two authors independently screened articles, extracted data and assessed methodological quality.

**Results:**

From 2001 studies, 23 publications describing 19 different PROMs were included for analysis. Eight of these instruments were specific to the pelvic floor; four were only specific to pain and used across multiple disorders; three were generic quality of life instruments and four were other non-validated instruments such as focus group interviews. These instruments were not specific to pain in women with POP or SUI, as they did not identify all relevant domains such as the sensation, region and duration of pain, or incidents where onset of pain occurs.

**Conclusions:**

The findings of this review suggest there are no current PROMs that are suitable pain-specific instruments for women with POP or SUI. This knowledge may inform and assist in the development of a new PROM to be implemented into the APFPR.

**Supplementary Information:**

The online version contains supplementary material available at 10.1007/s00192-022-05126-4.

## Introduction

Pelvic floor disorders (PFDs) involve dysfunction of the muscles within the pelvic floor, where the pelvic muscles weaken or tighten leading to complications [1]. These complications can include stress urinary incontinence (SUI) and pelvic organ prolapse (POP). The International Urogynecological Association (IUGA) and International Continence Society (ICS) defines POP as the descent of one or more of the anterior vaginal wall, posterior vaginal wall, uterus or apex of the vagina [2]. In addition, SUI refers to the involuntary loss of urine on effort or physical exertion [2]. In Australia, up to 50% of women are affected by SUI and 9% are symptomatic for POP [3], with a 19% lifetime risk of requiring a pelvic floor reconstructive procedure [4]. Until recently, of the surgical interventions for SUI and POP, it was estimated that approximately 25% involve the use of a mesh product with an estimated 150,000 mesh devices being implanted in Australia since 1998 [5].

A number of women have reported adverse events such as chronic pain and erosion of mesh into the vagina [6] in response to undergoing pelvic floor surgical procedures involving transvaginal mesh implants. Women with SUI, and those that have complications following surgery for this disorder, have significantly poorer health-related quality of life (HrQoL) than their counterparts without SUI and pain due to surgery. As HrQoL is subject to the patients’ experience and personal beliefs, it is best described by patients themselves through patient-reported outcome measures (PROMs) [7].

A PROM is defined by the US Federal Drug Administration (FDA) as a “measurement of patient health status elicited directly from the patient” [7]. Many PROMs have been developed to measure HrQoL and can be either generic or specific to a condition, covering several specific domains such as fatigue, depression and pain [8, 9]. Registries are a proficient means of collecting disease-related PROMs as they routinely accumulate data from a large group of patients and thus can evaluate specified outcomes for a population [10]. The Australasian Pelvic Floor Procedure Registry (APFPR) was established in 2019 following a Senate inquiry into complications surrounding pelvic floor procedures that included pain and erosion of mesh into the vagina [11]. Due to the sometimes distressing and complex experience of pain from PFDs or complications associated with POP and SUI surgery, PROMs that measure an array of pain domains by capturing the type and range experienced in these circumstances can support early identification of relevant pain and the clinical management of patients undergoing these procedures [12]. The registry, which aims to provide support to women to report health outcomes regarding POP and SUI, would therefore benefit immensely from the inclusion of a pain-specific PROM.

Following an acceptability study conducted by the APFPR of PROMs in women following procedures for POP and SUI, it was found that women did not believe that current pain instruments were suitable for the registry [13]. Current PROMs from this study failed to recognize the sensation, region or duration of pain, or incidents where onset of pain occurs in women treated surgically for POP or SUI. While existing PROMs may have aspects that are relevant, there is not yet an instrument that covers all of these domains or where all questions are relevant for these groups of patients. Pain following surgery for POP or SUI is complex as it can exist for a variety of reasons including patient-related factors, the underlying conditions of the disorders, post-operative healing or a range of post-surgical complications including mesh exposure, infection, urinary retention and nerve injury [14]. A greater understanding and analysis of the pain may point to the underlying pathophysiology of this symptom, leading to further clinical investigation and appropriate health service management of the underlying cause [15].

The aim of this study was to review the existing literature and to identify whether there is a current PROM that measures pain in adult women suffering from POP or SUI for inclusion in the APFPR, which specifically reports complications from these two conditions.

## Materials and methods

**Fig. 1:**
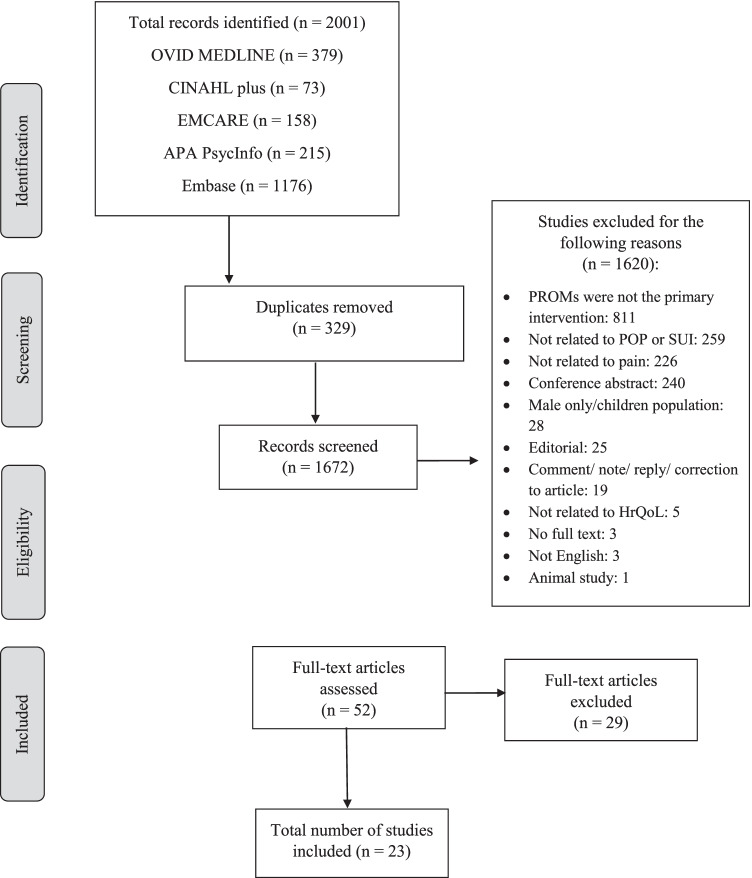
Preferred Reporting Items for Systematic review and Meta-Analysis (PRISMA) flowchart showing selection of articles for review

This systematic review was performed following the Preferred Reporting Items for Systematic review and Meta-Analysis (PRISMA) guidelines [16]. The databases searched include OVID MEDLINE, Embase, CINAHL, PsycINFO and EMCARE from inception to April 2021. Google Scholar was also searched as grey literature, but no additional papers were found. This review was registered on PROSPERO (ID: CRD42021250117). The initial MEDLINE search strategy included search terms “patient reported outcome measures” OR “patient health questionnaire” OR “self-report” OR “surveys” OR “questionnaires” OR “quality of life” OR “health related quality of life” OR “perception” AND “pelvic floor disorders” OR “pelvic floor dysfunction”. After the search strategy was finalized in MEDLINE, it was carried out in other databases and adapted as required using MeSH trees. The detailed search strategy is available as Supplementary material. The search was limited to the English language and human participants only (see Fig. 1).

### Eligibility criteria

We included quantitative and qualitative studies focusing on pain and PFDs involving POP and SUI. No restriction on year of publication was applied. Subjects were women, both inpatients and outpatients. Articles involving only male participants were excluded. Studies without a comparator were considered for inclusion. The main outcome of our analysis was to identify and evaluate all existing instruments used to measure pain in women with POP and SUI.

### Screening and selection

The first stage of screening involved two reviewers (MR, RR) reading titles and abstracts of all articles identified by the search. Any articles that clearly did not meet the inclusion criteria were removed. Exclusion criteria were studies where the article was not available in the English language as well as conference abstracts and editorials. Full texts of remaining articles were then read by two reviewers (MR, RR). The numbers of studies at each stage of the search were recorded using the PRISMA flow diagram.

### Data extraction

A data extraction form was constructed to summarize selected studies in line with the outcomes of the systematic review. The form was tested on a small number of articles and revised as necessary.

The following information was extracted:

- Type of study (cross-sectional, longitudinal, validation, development, review);
- Study population (number of participants, adults);
- Mean age of participants where provided;
- Setting in which PROM(s) administered (inpatient, outpatient, clinical trial);
- PROM(s) used;
- Type of PROM(s) (generic, specific);
- Time points PROM(s) administered (pre- or post-diagnosis, stage of study);
- Method of administration (interview, paper, online);
- Key findings of study.

A descriptive synthesis of results was undertaken, organized thematically by type, context, frequency, modes and methods of administration each measure.

The quality of the studies was assessed using the COnsensus-based Standards for the selection of health Measurement INstruments (COSMIN) risk of bias checklist [17]. The COSMIN tool was chosen as it is specifically designed for studies using PROMs. This tool includes assessment of ten domains, and each category was classified as very good, adequate, doubtful or inadequate, if applicable. Results are summarized into a table presenting the lowest score for each property [17].

## Results

### Search results

The search yielded 2001 results. After duplicates were deleted, 1672 articles remained. Studies were screened in two phases. An initial screen of titles and abstracts was conducted by two reviewers (MR, RR), which identified 52 articles that fit the inclusion criteria. A further screen of full texts eliminated 29 articles that met the exclusion criteria.

The final number of studies included in the review was 23 articles. The numbers at each stage are outlined in Fig. 1.

### Risk of Bias

Two authors (MR, RR) independently assessed the risk of bias of each of the studies following the COSMIN checklist. Several papers in this review did not validate the instruments used in their studies and thus were not critically appraised. The quality of the results was assessed after data extraction and a full risk of bias table can be found in the Supplementary material.

The COSMIN criteria are used to discern whether the psychometric properties of PROMs have been evaluated using rigorous measures so that reviewers can evaluate the quality of the instrument. For example, the most evaluated property was reliability, where a majority (59%) of instruments scored as ‘adequate’, followed by 25% as ‘very good’. This suggests that most instruments that could be tested for reliability were consistent in their measurements of pain. The second most common property was hypothesis testing for construct validity, where 90% of the eligible instruments scored as ‘adequate’. This suggests that most PROMs assessed were adequately consistent with hypotheses based on the assumption that the PROM validly measures the construct to be measured.

### General findings

Twenty-three full texts were included in analysis. Studies were undertaken from 1998 to 2020, with the majority (*n* = 18, 78%) published between 2011 and 2020 and 22% (*n* = 5) published between 1998 and 2010. All studies analysed PFDs, with 43% (*n* = 10) specifically reporting on SUI [18–27] and 13% (*n* = 3) focusing solely on POP [28–30]. Three studies included patient groups of women post-birth [26, 29, 31], and four studies recruited women who would have or had surgery for a POP or SUI [30, 32–34]. Most (*n* = 7, 30%) studies were conducted in the USA [21, 30, 32, 35–38]. There were three (13%) full texts published in Europe [22, 31, 39], three (13%) in the UK [20, 29, 40] and three (13%) in South America [23, 24, 26]. Other studies (*n* = 3, 13%) were conducted across multiple nations [18, 19, 34] or in Asia (n = 3, 13%) [27, 41] and one (4%) in Canada [33].

### PROMs identified

We identified 19 different PROMs that focussed on pain across 23 full-text articles included in this review (see Table 1).

**Table 1:** Identified instruments and how they measured pain

Instrument	How pain was measured
Pain specific	
BPI	Pain (not specific to PFDs)
Pain Catastrophizing Scale (PCS)	Pain (not specific to PFDs)
Short Form McGill Pain Questionnaire (SFMPQ)	Post-operative pain (not specific to PFDs)
VAS	Residual pain, mean pain intensity, perceived suffering (not specific to PFDs)
Condition specific	
ePAQ-PF	Vaginal pain, bladder pain, pain relieved by micturition, dragging pain, pain and sex
FSFI	Pain during sexual intercourse, rating of pain
GUPI	Pain at entrance to vagina, pain in the vagina, pain in the urethra, pain during or after sexual intercourse
ICIQ (ICIQ-VS, ICIQ-UI-SF)	ICIQ-VS: awareness of dragging pain in lower abdomen
LURN SI-29	Bladder pain
PFDI (PFDI-20, PFDIQ-SF20)	Pain/discomfort in the lower abdomen or genital region
PISQ (PISQ 12, PISQ-IR)	Pain during sexual intercourse, pain stopping one from being sexually active
UDI-6	Pain, pain in lower abdominal/genital area
Generic	
EQ-5D-5L	Pain, pain/discomfort (not specific to PFDs)
KHQ	Sensation of pain, pain in body part (not specific to PFDs)
SF-36	Bodily pain (not specific to PFDs)
Other	
Focus groups	Ranking of pain as an adverse effects post-surgery
Other non-validated questions	Expectation of post-operative pain, self-reported pain tolerance before and after surgery in both POP and SUI groups
Pain question	Pain as a patient symptom after surgery for POP
Semi structured interview	Pain reported as ‘pelvic floor problems’ post-partum

BPI: Brief Pain Inventory

ED-5D-5L: 5 level EuroQol 5

ePAQ-PF: the electronic Personal Assessment Questionnaire-Pelvic Floor

FSFI: Female Sexual Function Index

GUPI: Genitourinary Pain Index

ICIQ (-VS, -UI-SF): International Consultation on Incontinence Questionnaire (-Vaginal Symptoms, -Urinary Incontinence-Short Form)

KHQ: King’s Health Questionnaire

LURN SI-29: Lower Urinary Tract Dysfunction Research Network Symptom Index-29

PFDI (-20): Pelvic Floor Disability Index (20)

PFDIQ-SF20: Pelvic Floor Distress Inventory Questionnaire-Short Form 20

PISQ (-12, -IR): Pelvic Organ Prolapse Incontinence Sexual Questionnaire (12, IUGA-Revised)

SF-36: 36-Item Short Form Survey

UDI-6: Urinary Distress Inventory-6

VAS: visual analogue scale

Most (*n* = 12, 52%) of the studies reported both generic and specific instruments. The next most frequent were articles that contained only condition-specific PROMs (*n* = 8, 35%), followed by publications reporting generic instruments (*n* = 3, 13%). There was one study reporting a telephone survey [37], one semi-structured interview [42] and one study involving focus groups [32].

### Pain-specific instruments

There were four pain-specific instruments; however, none were targeted to the pelvic floor or related/referred pain. Pain-specific instruments were reported in four (17%) articles [22, 27, 33, 43]. The Brief Pain Inventory (BPI) was used once by Tincello et al. [43] to measure ‘post-operative pain’ on a scale of 0 (no) to 10 (severe). The Pain Catastrophizing Scale (PCS) was used once by Larouche et al. [33] to measure ‘pre-operative pain’ with a score of 0 to 52. The McGill Pain questionnaire measured ‘post-operative pain’ in the same article, ranging from 0 to 10 [33]. The visual analogue scale (VAS) was utilized in three (13%) studies [22, 27, 43] and thus was the most used pain-specific instrument in this systematic review. The VAS measured ‘pain’ on a scale of 0 (no pain) to 10 (pain as bad as it could be) [44] pre- [43], peri- and post-operatively [22] in women who underwent surgery for a PFD as well as in women who attended a urology or gynaeco-urology clinic for a PFD [27].

### PFD-specific instruments

Nearly half (42%) of the instruments identified in this review were condition-specific relating to POP or SUI. Most instruments covered just one area of pain, whether that was described as just ‘pain’ or ‘bodily pain’, for example; yet two, the electronic Personal Assessment Questionnaire-Pelvic Floor (ePAQ-PF) and the Genitourinary Pain Index (GUPI), covered an array of pain-related domains [45, 46]. Dua et al. [40] and Elenskaia et al. [29] utilized ePAQ-PF to measure vaginal pain, bladder pain, pain relieved by micturition, dragging pain and pain during or after sex. Cella et al. [21] validated the Lower Urinary Tract Dysfunction Research Network Symptom Index-29 (LURN SI-29) against GUPI, which measured pain at entrance to the vagina, pain in the vagina, pain in the urethra as well as pain during or after sexual intercourse. Different versions of the Pelvic Floor Distress Inventory (PFDI), including the Pelvic Floor Distress Inventory Questionnaire-Short Form 20 (PFDIQ-SF20) and the Pelvic Floor Disability Index-20 (PFDI-20), were analysed in five different articles for “pain or discomfort in the lower abdomen or genital region” [21, 30, 32, 33, 41]. In fact, five instruments across ten studies measured some sort of pain in the abdominal, vaginal or genital region [21, 28–30, 32, 33, 37, 40, 41, 47]. Four PROMs [ePAQ-PF, GUPI, the Pelvic Organ Prolapse/Urinary Incontinence Sexual Questionnaire (PISQ) and the Female Sexual Function Index (FSFI)] were used to assess pain during intercourse across multiple articles [21, 24, 29, 30, 35, 40, 48] and two instruments (ePAQ-PF, LURN SI-29) measured bladder pain specifically [21, 29, 40]. URIS-24 [36] did not actually measure pain itself but was validated against the pain domain of the SF-36. Furthermore, ICIQ-UI-SF version of the ICIQ tool did not measure pelvic floor pain; however, the ICIQ-VS did, measuring “awareness of dragging pain in lower abdomen” [28].

### Generic instruments

Three different generic HrQoL instruments were identified across multiple articles. The five-level EuroQol 5 (EQ-5D-5 L) was used by Tincello et al. [43] and Cashman et al. [49], utilizing one of the five domains in the instrument to measure pain in a “non-specific manner” prior to surgery and 3 months post-surgery. ‘Pain/discomfort’ associated with urinary incontinence in women was also measured by Dayana et al. [24] using the EQ-5D-5L. Both Dayana et al. [24] and Leroy et al. [26] included the King’s Health Questionnaire (KHQ) in their studies, with no mention of a pain domain, but compared this instrument to another HrQoL instrument, the Medical Outcomes Study 36-Item Short Form Health Survey (SF-36), which does have a pain domain. SF36 was utilized in 26% (*n* = 6) of studies to measure bodily pain [19, 24, 26, 27, 30, 41] with the questions: “how much bodily pain have you had during the past 4 weeks?” and “how much did pain interfere with your normal work?” [50].

### Other non-validated questions to measure pain

Four papers described other instruments and means to assess pain in women with PFDs, including semi-structured interviews and focus groups. Buurman et al. [42] utilized a semi-structured interview in women 1 month and 1 year post-birth to discuss perception of PFDs, where all women (*n* = 26) reported pain, including pain that they were not anticipating. Dunivan et al. [32] utilized focus groups to rank adverse effects, one of which included pain that was ‘very severe’, ‘moderately to somewhat severe’ or ‘not severe’. Furthermore, Larouche et al. [33] used non-validated questions that addressed post-operative pain, and LeBrun et al. [30] described the inclusion of ‘patient reported symptoms of pain’ after surgery for POP in the Pelvic Floor Disorders Registry (PFDR).

## Discussion

### General findings

This was the first systematic review to look at PROMs that measure pain in women with POP or SUI. We found that there were no validated condition-specific instruments that incorporated all of sensation, region and duration of pain, and that could capture all clinical scenarios where onset of pain occurs in women who suffer from PFDs and their related surgical complications. PROMs were solely PFD-specific instruments (but not focused on pain as a symptom) [21, 26, 28, 29, 35–37, 40, 47, 48, 51], purely pain-specific instruments (these were general and not created with this population in mind) [22, 33], generic instruments (lacking specificity to both pain and women with PFDs) [19, 23, 24, 27, 43, 49, 51] or non-validated questions (which could not be standardized to measure pain in another population group) [30, 32, 33, 42]. There are a range of pain types that women with POP or SUI may experience, including pre-operative pain due to hypertonic pelvic floor/myalgia related to the underlying disorder, typical post-operative pain, atypical post-operative pain due to a surgical complication such as an infection or injury, and longer term pain due to pelvic mesh extrusion or breakdown [52]. The instruments found in this literature review did not capture all of sensation of pain, the region in the body where the pain culminates, how long it lasts or with what activities the pain onset occurs. These aspects of pain are important as they may suggest specific underlying pathophysiology worthy of further investigation as well as providing a more holistic understanding of the impact of the pain on women’s HrQoL.

### Pain-specific PROMs

Pain-specific instruments are not suitable to measure pain in women with PFDs as they are not targeted sufficiently to the unique range of issues and pain due to complications that this population can be affected by. The VAS [22, 27, 43] is a universal pain assessment tool and measured pain from 0 (none) to 10 (‘worst pain possible’) [53]. In addition, the McGill Pain Questionnaire measured ‘post-operative pain’ ranging from 0 to 10 [33]. The BPI was another instrument measuring post-operative pain on a scale of 0 (no) to 10 (severe) at discharge home or 24 h after surgery [43]. Furthermore, the PCS measured ‘pre-operative pain’ with a score of 0 to 52 [33]. Despite allowing for a quick assessment of both acute and chronic pain, the mono-dimensional aspect of these instruments may not be appropriate in revealing the quality of the painful experience or differentiating the types of pain that come with these conditions, or as a result of mesh procedure complications [54]. This could include painful voiding, mesh-related infection or severe vaginal pain aggravated by movements [14]. Additional qualitative descriptions of pain would increase the utility of these instruments, and furthermore, a better understanding of these pain characteristics may aid improvements in managing underlying causes of pain.

### PFD-specific PROMs

The UDI-6 instrument, which is condition specific and used for both POP and SUI, both pre- and post-surgery [37, 47], asks the question, “Do you experience pain or discomfort in your lower abdominal, pelvic or genital region?” with a ranking of 0 (not at all) to 3 (greatly). This question is suitable to women with PFDs as it targets a specific region of pain, however, fails to recognize different types of pain, as women with these conditions suffer from pressure or heaviness deep in the pelvic area to severe, sharp pains and cramping [55]. An instrument such as the UDI-6, gathering data that one “has pain”, is not descriptive enough to inform a health professional about pain type [56]. Other PFD-specific instruments were also not suitable to measure pain in women with PFDs. The ePAQ-PF, despite measuring pain in pelvic floor disorders, consists of 120 questions [29, 40]. The length of this instrument may result in patient burden. The LURN SI-29 only explores the frequency and time points of bladder pain, failing to uncover the nature and intensity of such pain [21]. Conversely, the GUPI assesses bladder pain symptoms, yet not the onset of such [21]. In addition, the PFDI, where versions were included in five studies [21, 30, 33, 38, 41], includes questions such as “Do you usually experience heaviness or dullness in the pelvic area?” with a scale of ‘no’ or ‘yes’ and, if yes, a pain rating of 1 to 4. In addition, the ICIQ-VS [28] asks “Are you aware of dragging pain in your lower abdomen?” with scores of 0 (never) to 4 (all the time). Ultimately, while questions like these are specific and more targeted to the population, they fail to retrieve information such as when the heaviness, dullness or dragging pain is felt, with what activities, whether the pain is constant or intermittent and when it first started. These type of ad hoc questions regarding pain do not truly capture the entirety of the pain.

### Generic PROMs

The generic instruments entailed questions that were rather broad, for example, in the SF-36: “How much bodily pain have you had during the past 4 weeks?” with a rating of ‘none’ to ‘very severe’ [50]. In addition, the EQ-5D-5L [24, 43, 49] asks patients to tick a box about their pain, where having no pain, slight pain, moderate pain, severe pain or extreme pain or discomfort are options. These pain questions may not be suitable for women with PFDs as ‘pain or discomfort’ in the ‘bodily’ region is not specific enough and does not inform us of the true sensations of pain. The quality and degree of pain are imperative as complications from procedures may be identified as a source of the patient’s pain [57]. Thus, generic PROMs that have pain domains may not be able to capture the full extent of pain suffered by women living with this condition and complications post-surgery [58].

### Other non-validated questions to measure pain

Moreover, non-validated questions regarding pain may better encapsulate a patient’s personal experience with pelvic floor-related pain. Dunivan et al. [32] incorporated the patients’ perspective utilizing focus groups at three separate surgery sites to discuss adverse effects, one including pain. A woman mentioned: “I have pain as well in my rectum. It feels like it gets pinched or something” [32]. The ability to converse with these women, compared to ticking a box in a questionnaire, is a benefit as the health professional can further deduce the true sensation, duration and region of pain, and the incidents where onset of pain occurs in women with PFDs. However, questions within focus groups and other semi-structured interviews are non-validated and therefore may not be reliable or applicable across other groups [59] as they are not standardized. A new validated PROM may be able to flag underlying clinical issues, whereby clinicians can further investigate through patient-specific consultation.

### Inclusion of pain instrument in the APFPR

Following review of the available pain questionnaires by clinicians and consumers, it was considered that given the significance of pain as a potential indicator of pathophysiology, as well as its impact on women’s HrQoL, there is a need for a new pain-specific PROM in the APFPR for women with POP or SUI. This review of the literature has confirmed that existing validated tools do not meet this need. The inclusion of a pain-related PROM into the APFPR will allow for further investigation of pain, especially as a complication post-surgery, and thus a more nuanced understanding of the impact of the pain on a woman’s HrQoL. Consequently, a new PROM developed for and included in the APFPR focusing on accurately measuring pain for POP and SUI could improve the quality of care and QoL of women living with these disorders. The development of a new PROM could be achieved through focus group questions and semi-structured interviews, providing a more personal insight into the woman’s experience and their subsequent HrQoL. A validated instrument created from these more ‘conversational’ type questions would provide huge benefit to the registry. However, a questionnaire that incorporates all pain types found in our search of the literature may be rather extensive. Therefore, it is very important for well conducted semi-structured interviews with women to highlight the most imperative pain types and time points. To do this, one method may be to conduct such interviews with both patients and pelvic floor clinicians and ask them what they deem to be relevant [60]. Furthermore, through qualitative interviews with women who suffer from PFDs themselves, content validity of the PROM may be deduced [60].

### Strengths and limitations

This systematic review synthesized data from five databases and thus produces a rather robust body of evidence. It utilized systematic methods to assess study quality. In addition, this review is the first to critically evaluate the types of pain instruments and their subsequent properties in women with POP or SUI. However, this systematic review has a limitations, that of being restricted to English language publications only, where other languages may have provided different insights into pain measurements using PROMs. A further search of grey literature and exterior databases could have been beneficial to include a wider variety of studies.

## Conclusion

This review aimed to identify whether there is a PROM that measures pain specifically for women with POP and SUI for inclusion in the APFPR, which specifically reports complications from these two conditions. We did not find a suitable pain-specific PROM designed for this population, and thus there remains a serious lack of substantial reporting on the HrQoL in women who continue to suffer pain following pelvic floor surgery. Based on a systematic review of the current literature, we suggest that the next step entails the development of a new instrument for pain, especially pain related to complications due to pelvic floor surgery, and one that will be suitable for inclusion into the APFPR. This new PROM may be suited for both pre- and post-surgery data collection.

## Supplementary Information

ESM 1 (DOCX 35 kb)
