# Supplementary material for: Patient-reported outcome measures for pain in women with pelvic floor disorders: a systematic review
Source: Int Urogynecol J. 2022 Mar 2;33(9):2325–34. doi: 10.1007/s00192-022-05126-4 (PMC9427903; doi:10.1007/s00192-022-05126-4)
Supplement: Supplementary file 1 — (DOCX 35 kb) [file 192_2022_5126_MOESM1_ESM.docx]

**Supplementary Material 1:** Complete Search Strategy

| **Database** | **OVID MEDLINE** |
| --- | --- |
| **Strategy** | **#1 OR #2 AND #3**  Limit to humans and English language |
| #1 | Patient reported outcome measures OR patient health questionnaire OR self-report OR surveys and questionnaires |
| #2 | Quality of life OR Health related quality of life.mp. OR Perception.mp. |
| #3 | Pelvic floor disorders OR pelvic floor dysfunction.mp. |
| **Database** | **PsycINFO** |
| **Strategy** | **#1 OR #2 AND #3**  Limit to humans and English language |
| #1 | Self-Report OR Patient Reported Outcome Measures OR Questionnaires OR Quality of Life Measures |
| #2 | Quality of Life OR Health Related Quality of Life OR Perception.mp. |
| #3 | Urinary Incontinence OR pelvic floor disorder.mp. OR prolapse.mp. |
| **Database** | **CINAHL plus** |
| **Strategy** | **#1 OR #2 AND #3**  Limit to humans and English language |
| #1 | Patient-Reported Outcomes OR self-report OR questionnaire |
| #2 | Quality of Life OR Perception OR health related quality of life |
| #3 | Pelvic floor disorders |
| **Database** | **EMCARE** |
| **Strategy** | **#1 OR #2 AND #3**  Limit to humans and English language |
| #1 | Patient-reported outcome OR self-report.mp. OR Questionnaire.mp. |
| #2 | Quality of life OR Perception.mp. |
| #3 | Pelvic floor disorder |
| **Database** | **Embase** |
| **Strategy** | **#1 OR #2 AND #3**  Limit to humans and English language |
| #1 | Patient-reported outcome OR Self report OR Questionnaire.mp. |
| #2 | Quality of life OR Health related quality of life.mp. OR perception.mp. |
| #3 | Pelvic floor disorder |

| **Supplementary Material 2:** Data Extraction | | | | | | | | | | | | |
| --- | --- | --- | --- | --- | --- | --- | --- | --- | --- | --- | --- | --- |
| **Author, year**  **Country,**  **Study type** | **Setting** | **Aims** | **Patient group** | **Age mean** | **Population size (n)** | **Developed instrument** | **If new - development** | **Type of PROM** | **Why PROM used?** | **Outcomes measured** | **Method of administration** | **Time points** |
| Bordeianou et al, 2020  Multinational  Review | PFD Consortium: clinicians with expertise in care of PFD | To generate inclusive guidelines for the practice of PFDs | Patients with PFD | Not stated | Not stated | NA | IMPACT form, includes UDI-6 | Specific | To assess urine leakage, difficulty emptying and pain | UI symptoms | Questionnaire | NA |
| Bushnell et al, 2005  Multinational  RCT | 4 clinical trial settings and 2 clinic (population) settings | To report the psychometric performance of the I-QOL tool in 15 languages | Women with SUI of at least 3 months | 52.2 | 1919 | SF-36 | NA | Generic | To measure domain of bodily pain | Bodily pain | Questionnaire | Baseline and 2 weeks for RCTs, and 2 or 3 weeks in communities |
|  |  |  |  |  |  | I-QOL |  | Specific | I-QOL: no mention of pain measurements in this paper |  |  |  |
| Buurman et al, 2013  The Netherlands  Observational | Family practitioners' populations | To explore women's perception of PFD after birth | Women post birth (1mo-1y) | Majority age range was 30-35 | 26 women | NA | Semi structured, in-depth interviews | Generic | To assess women with PFDs’ perception | Pain | Semi-structured interview | 1 month and 1 year after vaginal delivery |
| Cashman et al, 2018  UK  Retrospective | All UK urologist surgeons for SUI eligible to submit data | To summarise surgical management of females over a 3-year period | Women having procedures for SUI | 54 | 2917 procedures | EQ-5D- 5L | NA | Generic | EQ-5D-5L measured pain | Pain pre- and post- op | Questionnaire | Baseline and 3 months |
|  |  |  |  |  |  | ICIQ-UI-SF |  | Specific | ICIQ-UI-SF: mention of pain in this paper |  |  |  |
| Cella et al, 2019  USA  Validation study | Academic medical centres | To develop a new PROM: LURN SI-29 | Men and women with LUTS | 60 | 150 women | NA | LURN SI-29 | Specific | To compare against pain domains of others: GUPI, PFDI-20 | Bladder pain, vaginal pain | Questionnaire | Baseline, 3 and 12 months |
| Chan et al, 2017  Hong Kong  Validation study | Urogynaecology clinic | To investigate the reliability and validity of Chinese version of the PFDI and PFIQ | Women with PFD | 55 | 597 women | PFDI | NA | Specific | To compare pain domain (PFIQ: no mention of pain measurement in this paper) | Pain | Questionnaire | Week 0, week 4 |
|  |  |  |  |  |  | SF-36 |  | Generic | To compare 'bodily pain' domain to PFDI & PFIQ |  |  |  |
| Collinet et al, 2008  France  Observational | Hospitals in France | To generate safety data on of patients undergoing surgery for TVT-O | Women with symptoms of SUI | 57.5 (range 27-93 years) | 984 | VAS | NA | Generic | To report perioperative or post-operative pain complications | Pain | Questionnaire | At first post-op visit, between 4 and 12 weeks after |
| Constantine et al, 2016  USA  Validation study | 3 clinical sites | To improve upon PISQ-IR and evaluate potential PFD impact on women | Women seeking care for PFDs | 54.9 for PISQ-IR | 141 women phase 1, 1589 phase 2 | PISQ-IR | NA | Specific | To measure pain in different criterion measures | Pain | Questionnaire | Baseline, 6 months |
| Dantas et al, 2019  Brazil  Validation study | Not stated | To link the content of 4 most used questionnaires to assess QoL of women with UI, with the ICF | No patient group | Not stated | Not stated | KHQ | NA | Generic | To compare against ICF category description of pain | No mention of pain measurement by these PROMs in this paper | Questionnaire | No time points |
|  |  |  |  |  |  | ICIQ, I-QOL, BFLUTS |  | Specific |  |  |  |  |
| Dayana et al, 2017  Brazil  Cross sectional | Two outpatient clinics of urogynaecology | To identify the most frequent type of UI in women and compare QoL | Women with different types of UI | Mean age for SUI 49, age range 22 to 89 | 556 women | SF 36, KHQ, EQ-5D | NA | Generic | EQ-5D-5L: pain, SF-36: bodily pain, KHQ: no mention of pain in this paper | Pain | Questionnaire | At entry |
|  |  |  |  |  |  | ICIQ-UI-SF, PISQ-12 |  | Specific | No mention of measuring pain in this paper |  |  |  |
| Dua et al, 2014  UK  Retrospective | Women that attended urogynaecology clinics in 3 years | To evaluate relationship between age and the impact of PFDs using ePAQ-PF | Women with PFDs | Majority b/w 51-65 years | 4311 women | ePAQ-PF | NA | Specific version | To measure vaginal pain, bladder pain | Pain | e- Questionnaire | Data collected between June 2007- March 2010 |
| Du Beau et al, 1999  USA  Validation study | University affiliated community-based practice and tertiary hospital | To develop a PROM for assessing the impact of urge UI on QoL | Older persons with UI | Women 67.7 | UI assessed in 24 women | NA | URIS-32 | Specific | To measure QoL in older persons with UI | No pain measurement mentioned, but tested for validity against SF-36 | Questionnaire (43 mailed, 3 in-person) | Two occasions one week apart |
| Dugan et al, 1998  USA  RCT | Primary care practices | To determine the importance of QoL of older adults | Adults > 60y visiting a primary care practice | 72.5 | 435 UI, 384 females | UDI-6 and IIQ-7 | NA | Specific | To measure QoL | UDI-6: pain in lower abdominal or genital area, other PROMs did not mention pain in this paper | Telephone survey | At entry |
|  |  |  |  |  |  | GLS |  | Generic |  |  |  |  |
| Dunivan et al, 2017  USA  Cross sectional | 3 separate surgery groups: pre-op, short term post-op, long-term post-op | To describe patient perceptions on adverse events from the provider's perspective | Women that will get/ have had surgery for pelvic floor disorders | 58.4 | 81 women | NA | Focus groups | Specific | To assess patient understanding and perspective | Pain assessed through focus group ranking of adverse effects | Focus groups at different sites & questionnaire | At entry |
|  |  |  |  |  |  |  |  | Specific |  |  |  |  |
| Ekanayake et al, 2017  Sri Lanka  Validation study | Two gynaecology units | To translate and validate this tool into Sinhala and Tamil | Gynaecology units of hospitals - women presenting with and without POP | ▪Sinhala w POP 56.2, w/o 42.6 ▪Tamil w POP 60.4, w/o 41.4 | 40 women for each language | ICIQ- VS | NA | Specific | To assess pain | Awareness of dragging pain in lower abdomen | Questionnaire | Week 0, week 4, if they had surgery then week 8 |
| Elenskaia et al, 2012  England  Cohort study | University Hospital | To evaluate pelvic floor symptoms and effect on QoL during pregnancy | Women with uncomplicated singleton pregnancy | 29.7 | 148 women | ePAQ-PF | NA | Specific | To measure pain in bladder and bowel and assess sex and vaginal symptoms | Bladder pain, pain relieved by micturition, dragging pain, pain and sex | Questionnaire | In the 2nd trimester (20 weeks) and then at 36 weeks gestation |
| Grzybowska et al, 2019  Poland  Cross sectional | Gynaecology clinic | To determine cut-off scores for sexual dysfunction in PISQ-IR for women with PFDs | Women with PFD | 56.6 | 226 women | PISQ-IR, FSFI | NA | Specific | To measure pain using FSFI,  PISQ-IR: no mention of pain measurement in this paper | Pain | Questionnaire | At entry |
| Habashy et al, 2019  USA  Review | Not stated | To examine the use of PROMs in the evaluation of PFD | No patient group | Not stated | Not stated | SPEQ | NA | Generic | To assess sexual dysfunction | No mention of how these PROMs measure pain in this paper | Questionnaire | Not stated |
|  |  |  |  |  |  | ICIQ, PFDI, PFIQ, PISQ |  | Specific | To compare the uses and benefits of using different PROMs |  |  |  |
| Larouche et al, 2020  Canada  Observational | Inpatient urogynaecologic surgery cohort | To explore correlation between perioperative symptoms of depression/ anxiety after PFD surgery | Women undergoing surgery for PFD | 58.5 | 60 women | PCS, SFMPQ, 'other non-validated questions', PFDI, PFIQ | NA | Generic | To measure pain before and after surgery | Pain | Questionnaire | Baseline, post-operation |
| LeBrun et al, 2016  USA  Observational | Collaboration between industry, surgeons and other clinicians caring for POP | To give evidence for best medical practices for treating patients with PFD | Women with PFD: specifically POP | Not stated | Not stated | PFDI-20, PFIQ-7, PISQ-12, ICIQ-UI-SF | NA | Specific | To provide correlation between QOL and measures of treatment outcomes | Pain measured as patient symptom by provider in PFDR (not these PROMs) | e-Questionnaire | Baseline, 2, 6, 12, 18, 24, 36 mo. |
| Leroy et al, 2012  Brazil  Observational | Obstetrics Outpatient Clinic of a public teaching hospital | To evaluate whether/how UI in the puerperium compromises HrQoL | Women up to 90 days post-partum | 25.9 | 344 women | ICIQ-UI-SF | NA | Specific | Pain in case and control group | ICIQ-UI-SF: no mention of pain measurement in this paper | Questionnaire | At entry |
| Margalith et al, 2004  Israel  Cross-sectional l | Urology or gynaeco-urology clinic at two university medical centres | To describe QoL, stress and patterns of seeking care among women with SUI | Women attending clinics with SUI | 49 | 131 women | SF 36, VAS | NA | Generic | Bodily pain | Pain measurement | Questionnaire | At entry |
| Tincello et al, 2011  Multinational  Cohort study | 29 centres over 2 years | To examine effectiveness of a single incision sling | Patients that had surgery using single incision, retro pubic or obturator sling | Not stated | 1398 treated, post op data available on 1334 | I-QOL | NA | Specific | I-QOL: no mention of pain measurement in this paper | Pain | Questionnaire | Pre-op, at each post-op visit (baseline and 12 mo.) |
|  |  |  |  |  |  | EQ-5D-5L, BPI |  | Generic | EQ-5D-5L measures pain using VAS, BPI measures pain |  |  |  |

| BFLUTS: Bristol Female Lower Urinary Tract Symptoms  BPI: Brief Pain Inventory  ED-5D-5L: 5 level EuroQol 5  ePAQ- PF: the electronic Personal Assessment Questionnaire – Pelvic Floor  FSFI: Female Sexual Function Index  GLS: Global Life Satisfaction  GUPI: Genitourinary Pain Index  HrQoL: Health-related Quality of Life  ICF: International Classification of Functioning, Disability and Health  ICIQ (-VS, -UI-SF): International Consultation on Incontinence Questionnaire (-Vaginal Symptoms, -Urinary Incontinence – Short Form)  IIQ-7: Incontinence Impact Questionnaire Short Form  IMPACT: Initial Measurement of Patient-Reported Pelvic Floor Complaints  I-QOL: Incontinence Quality of Life Survey  KHQ: King’s Health Questionnaire  LURN SI-29: Lower Urinary Tract Dysfunction Research Network Symptom Index- 29  LUTS: Lower Urinary Tract Symptoms  OAB: Overactive Bladder | PCS: Pain Catastrophizing Scale  PFD: Pelvic Floor Disorder  PFDI (-20): Pelvic Floor Disability Index (20)  PFDR: Pelvic Floor Disorder Registry  PFIQ: Pelvic Floor Impact Questionnaire  PISQ (-12, -IR): Pelvic Organ Prolapse Incontinence Sexual Questionnaire (12, IUGA-Revised)  PROM: Patient Reported Outcome Measure  QoL: Quality of Life  RCT: Randomised Control Trial  SF-36: 36- Item Short Form Survey  SFMPQ: Short Form McGill Pain Questionnaire  SPEQ: Short Form Personal Experiences Questionnaire  SUI: Stress Urinary Incontinence  TVT-O: Transvaginal Tape  UDI-6: Urinary Distress Inventory- 6  UI: Urinary Incontinence  URIS-32: 32-item Urge Impact Scale  VAS: Visual Analogue Scale |
| --- | --- |

## **Supplementary Material 3:** Risk of Bias

|  | **1. PROM development** | **2. Content validity** | **3. Structural validity** | **4. Internal consistency** | **5. Cross cultural validity** | **6. Reliability** | **7. Measurement Error** | **8. Criterion validity** | **9. Hypothesis testing for construct validity** | **10. Responsiveness** |
| --- | --- | --- | --- | --- | --- | --- | --- | --- | --- | --- |
| **FSFI** | | | | | | | | | | |
| Grzybowska 2019 | - | Very good | - | - | Adequate | - | Doubtful | Doubtful | Adequate | Adequate |
| **GUPI** | - | - | - | - | - | - | - | - | - | - |
| Cella 2019 | Very good | - | - | Adequate | - | Adequate | - | - | - | Adequate |
| **ICIQ** | - | - | - | - | - | - | - | - | - | - |
| Ekanayake 2017 | Very good | Very good | - | Very good | Adequate | Very good | - | - | - | Very good |
| **LURN SI-29** | - | - | - | - | - | - | - | - | - | - |
| Cella 2019 | Very good | Adequate | Doubtful | - | Adequate | - | - | - | Adequate | - |
| **PFDI** | | | | | | | | | | |
| Chan 2017 | Very good | - | - | Very good | - | Adequate | - | - | Adequate | - |
| **PISQ** | | | | | | | | | | |
| Constantine 2016 | Very good | - | - | - | Very good | Adequate | - | Adequate | - | - |
| Grzybowska 2019 | - | - | - | - | - | - | - | - | - | - |
| **UDI- 6** | | | | | | | | | | |
| Bordeianou 2020 | - | Adequate | Adequate | Doubtful | - | Adequate | Adequate | - | Adequate | Adequate |
| **VAS** | | | | | | | | | | |
| Chan 2016 | - | - | - | - | - | - | - | - | - | - |
| **SF-36** | | | | | | | | | | |
| Bushnell 2005 | - | - | - | Very good | - | - | - | - | Adequate | - |
| Chan 2017 | - | - | - | - | - | - | - | - | - | - |
